# ﻿Molecular, morphological, and morphometric evidence reveal a new, critically endangered rattlepod (*Crotalaria*, Fabaceae/Leguminosae, Papilionoideae) from tropical China

**DOI:** 10.3897/phytokeys.242.122407

**Published:** 2024-06-11

**Authors:** Shabir A. Rather, Sirilak Radbouchoom, Kaikai Wang, Yunxue Xiao, Hongmei Liu, Harald Schneider

**Affiliations:** 1 Center for Integrative Conservation & Yunnan Key Laboratory for Conservation of Tropical Rainforests and Asian Elephants, Xishuangbanna Tropical Botanical Garden, Chinese Academy of Sciences, Mengla, Menglun 666303, Yunnan, China; 2 University of Chinese Academy of Sciences, Beijing 100049, China; 3 Center for Horticulture and Gardening & Yunnan Key Laboratory for Conservation of Tropical Rainforests and Asian Elephants, Xishuangbanna Tropical Botanical Garden, Chinese Academy of Sciences, Mengla, Menglun 666303, Yunnan, China

**Keywords:** Biodiversity, conservation, *Crotalaria*, endemism, Leguminosae, Xishuangbanna

## Abstract

Here, we describe a new species of *Crotalaria* L. discovered in Mengla County, Xishuangbanna Dai Autonomous Prefecture, Yunnan, China. The new species, *Crotalariamenglaensis* S.A.Rather, was confirmed by identifying diagnostic morphological characteristics, performing principal component analyses of phenotypic traits, and phylogenetic analyses based on nuclear ITS and plastid *mat*K sequences. Phylogenetic analyses recovered the two accessions of the new species to be sister to *C.bracteata* Roxb. ex DC. In turn, these two species formed the sister clade to the two accessions of *C.incana* L. The morphometric analyses revealed that all three species were distinct, while the analyses of distinctive characters enabled unambiguous distinction of the new species by its growth habit, leaflets, flower structure and pod morphology. In contrast to the two related species, the new species is currently known only from *ca.* 100 mature individuals. Thus, this species is considered to be critically endangered.

## ﻿Introduction

Xishuangbanna, located in the most southwestern part of Yunnan Province and sharing borders with Myanmar and Laos, is well recognized for its rich biodiversity. Its tropical forests play a vital role in global terrestrial biodiversity conservation efforts (Feng et al. 2018). Unfortunately, many plants in Xishuangbanna have recently faced significant threats from deforestation and the establishment of artificial plantations, especially rubber plantations (Brinck et al. 2017; Liu et al. 2017; Yang et al. 2021; Yang et al. 2023). To enable effective protection of the unique and rich diversity of Xishuangbanna, efforts are needed to record species diversity, including that of many species still unknown to science (Chang et al. 2018; Chang et al. 2022; Chen et al. 2022).

Here, we focus on accessions belonging to the legume genus *Crotalaria* L., which comprises approximately 702 species worldwide (Rockinger et al. 2017; Rather et al. 2018). Its highest species diversity is found in Africa and Madagascar, with an estimated 540 species. It has also expanded to South America and North America, with 64 and 34 species, respectively (Flores et al. 2006; Pandey et al. 2010; Le Roux et al. 2013; Rather et al. 2018). India hosts the largest number of species in Asia (ca. 80 species), followed by Southeast Asian countries, which collectively host 105 species (Lock and Simpson 1991; Rather et al. 2018). Approximately 45 species have been recorded to occur in China, predominantly in Southwest China, including nine endemics and six introduced species (Li et al. 2010). The genus exhibits both annual and perennial life forms and various growth forms (prostrate to erect herbs, undershrubs, robust shrubs, and occasionally small trees) and occupies various habitats, such as open grasslands, roadsides, and forest edges (Polhill 1982; Rather et al. 2018). *Crotalaria* is characterized by papilionoid flowers, the presence of paired callosities on the standard petal, a rostrate keel, 5 + 5 dimorphic anthers, a hairy style, inflated pods and the presence of pyrrolizidine alkaloids (Le Roux et al. 2013; Rather et al. 2018).

In the present study, several interesting specimens of *Crotalaria* were collected during field trips to Mengla County in Yunnan Province, China. Initially, some plants observed in the Mengpengzhen area of the Xishuangbanna Dai Autonomous Prefecture could not be assigned to any known taxa. Thus, we considered three priority explanations. The first explanation considered interpreted that these accessions are natural hybrids formed between two sympatrically occurring *Crotalaria* species, namely, *C.bracteata* Roxb. ex DC. and *C.incana* L. However, upon closer examination, the newly discovered species did not match either of these taxa. The plants exhibited differences in numerous characteristics, including plant height, leaflet shape, inflorescence, flower, pod shape, indumentum, and number of seeds per pod, among others. The subsequent discovery of numerous plants during further surveys, which included nearly 50 mature individuals and several immature plants spread over an area of 0.1 km^2^, eliminated the possibility that these plants were hybrids. The second explanation interpreted these accessions as a new distributional record of a known species within the genus *Crotalaria* L. However, there have been no documented new records for the genus *Crotalaria* L. This possibility was ruled out after unsuccessful attempts to identify the plants using existing identification keys (Brach and Song 2006; Li et al. 2010). Additionally, we consulted taxonomists at various institutes in China who were unable to recognize the taxa collected. Furthermore, comparisons with verified images of other *Crotalaria* L. taxa available in the Plant Image Library of China (PPBC; https://ppbc.iplant.cn/) also failed to yield any proper matches. The final explanation considered these plants to represent a new, previously undescribed taxon. This study was designed to confirm this hypothesis by focusing on three lines of evidence, namely, traditional diagnostic morphological character identification, morphometric studies using principal component analyses, and phylogenetic analyses using both plastid *mat*K and nrITS DNA sequences. Finally, we present a comprehensive taxonomic description of this newly discovered *Crotalaria* L. species, supplemented with taxonomic comments and accompanying photographs.

## ﻿Materials and methods

### ﻿Ethics statement

The geographic sites where the newly identified species was found do not coincide with any designated natural conservation areas. Therefore, specific permission for access to these locations was not needed.

### ﻿Morphological observations

The morphological analysis and description of the newly discovered species were prepared using freshly collected samples. The flowers were preserved in FAA solution (formaldehyde–glacial acetic acid–alcohol) for further studies. They were rehydrated using a mixture of water and detergent to observe the corolla in detail, followed by dissection. Minute corolla features were examined using a Stemi 305 binocular microscope. Morphological terminology adhered to the standards set by Harris and Harris (2001), Hickey and King (2007) for vegetative characters, Hewson (1990) for indumentum description, and Endress (2010) for inflorescence morphology. A comparison of the significant morphological features of the new species with those of its allied species *C.incana* L. and *C.bracteata* Roxb. ex DC. was performed (Table 1).

**Table 1.** Comparisons among *Crotalariamenglaensis* S.A.Rather, *C.incana* L. and *C.bracteata* Roxb. ex DC. The bold font represents the main distinguishing features of the new species.

Morphological characters	*Crotalariamenglaensis* S. A. Rather	*Crotalariaincana* L.	*Crotalariabracteata* Roxb. ex DC
**Habit**	**Stiff, erect herbs**	**Shrublets**	**Shrublets**
**Height**	**0.5 m**	**1 m**	**0.6–1.2 m**
**Stem surface**	**Pubescent with white hairs**	**Pubescent brownish**	**Densely pubescent, brownish-yellow hairs**
Petiole	23–39 mm	30–50 mm	30–50 mm
**Petiole surface**	**Pubescent with white hairs**	**Glabrous**	**Glabrous**
Stipule	Acicular	Acicular	Acicular
**Leaflet size**	**30–80 × 21–31 mm**	**20–40 × 10–20 mm**	**50–70(–90) × 25–40 mm**
**Leaflet shape**	**Ovate to oblanceolate**	**Elliptic obovate, obovate, or suborbicular**	**Narrowly elliptic**
Leaflet apex	Acute	Obtuse and mucronate	Acuminate
Leaflet base	Attenuate	Rounded to broadly cuneate	Attenuate
**Leaflet surface (abaxial)**	**Pubescent**	**Glabrous**	**Sparsely pilose**
**Leaflet margin**	**Puberulent entire margin**	**Simple and ciliate**	**Slightly involute and non ciliate**
**Bract shape**	**Lanceolate**	**Caducous**	**Acicular**
**Bract surface**	**Pilose**	**Glabrous**	**Glabrous**
Bract size	1.2–2.0 × 0.6–0.7 mm	1.5–2.2 × 0.4–0.7 mm	1–1.5 × 0.1–1 mm
**Bract position**	**Attached to the base of the pedicel**	**None**	**None**
**Bract number**	**One**	**None**	**None**
Bracteole size	2.7–3.1 × 1.6–1.8 mm	2–3 mm	1–2 mm
Bracteole surface	Hirsute	Pubescent	Pubescent
Bracteole margin	Entire	Slightly involute	Slightly involute
**Bracteole shape**	**Ovate to obovate with an asymmetrical base**	**Linear**	**Linear**
Inflorescence	Terminal or axillary raceme	Terminal or axillary raceme	Axillary raceme or rarely terminal
**Inflorescence length**	**80–120 mm terminal raceme; 110–170 mm axillary raceme**	**100–200 mm**	**100–150 mm**
**Number of flowers per inflorescence**	**Up to 12 terminal racemes; up to 47 axillary racemes**	**5–15**	**10–30**
Flower colour	Primrose or Strong pale yellow	Yellow	Yellow
Flower size	10–11.9 × 3.3–4 mm	10 × 5 mm	7–10 × 9 mm
Pedicel length	0.47 mm	0.3–0.4 mm	0.3–0.7 mm
Calyx length	5 mm	6–8 mm	5–6 mm
Calyx tube	2.4 mm	8.1 mm	7.6 mm
**Standard shape**	**Obovate-orbicular**	**Elliptic**	**Oblong**
Standard dorsal surface	Hispid at the middle and tomentose at the base	Pubescent	Pilose on the back at the apex
Standard size	88 × 74 mm	8–1 mm	9 mm
**Standard apex**	**Notched**	**Rounded**	**Rounded**
Standard Claw size	1.4 mm	6 mm	2.4 mm
**Callosity**	**Planar**	**Ridge**	**Ridge**
Wing size	7.1–7.3 × 2.3–2.9 mm	Staminal sheath ca. 1.5 mm long	8 mm
Wing claw size	1.52–1.84 × 6.3–0.77 mm	6–7.5 × 1–2.2 mm	2–2.7 × 1.1–0.9 mm
Keel size	10.1–15.1 × 4.8 mm	5.5–6.5 × 2.5–6 mm	8 mm
**Keel shape**	**Angled**	**Subangled**	**Subangled**
Keel alae	Present	Absent	Absent
**Keel curvature**	**Below middle**	**Lower third**	**Lower third**
**Keel vestiture**	**Glabrous**	**Lanate**	**Lanate**
**Keel beak**	**Straight**	**Spirally twisted up to 90**°	**Slightly incurved**
Keel claw	3.4–3.6 × 1.2–1.4 mm	4.5–4.7 × 1.5–1.7 mm	3.6–3.9 × 1.1–1.5 mm
Androecium size			
	Staminal sheath, 7.78 mm	Staminal sheath ca. 4.5 mm	Staminal sheath ca. 3.2–3.7 mm
	Filaments 1–1.3 mm long	Filaments 1.7–2.7 mm long	Filaments 5–2.9 mm long
	Longer anther, 1.2–1.5 mm	Longer anther, 1–1.4 mm	Longer anther, 1.2–1.7 mm
	Shorter anther, 0.5–0.6 mm	Shorter anther, 0.7–0.9 mm	Shorter anther, 0.8–0.9 mm
Gynoecium	Sub sessile	Sessile	Sessile
Gynoecium size	3.3 × 1.5 mm	2.1 × 1.5 mm	4.2 × 1.1–6 mm
**Style hairs**	**One row**	**All round**	**All round**
**Style bent from ovary/curved**	**Geniculate**	**Subgeniculate**	**Subgeniculate**
Pod stalk	4.63 mm	2 mm	7 mm
**Pod shape**	**Elliptic to oblong**	**Fusiform**	**Ellipsoid-fusiform**
Pod size	14.2–15 × 6–7.7 mm	20–30 × 6–7.7 mm	20 × 5–10 mm
**Pod indumentum**	**Tomentose**	**Rusty pilose**	**Densely rusty pubescent**
**Number of seeds per pod**	**12**	**20–30**	**7–8**
Seed size	2.2–2.5 × 0.9–1.2 mm	1.2–5 × 0.32–2 mm	1.8 × 5 mm
Seed colour	Bright citrine	Brown	Brownish black

The identification of the allied species *C.incana* L and *C.bracteata* Roxb. ex DC. was established through previous revisionary and systematic studies (Brach and Song 2006; Ansari 2008; Le Roux et al. 2013) and examinations of their types and other authentic specimens housed in large herbaria, such as PE, KIB, WUK, HITBC, CAL, DUH, FRLH, M, MH, SJC, and SKU. Additionally, virtual images of these species sourced from repositories such as the JSTOR Global Plants (JSTOR 2024), China Virtual Herbarium (Chinese Virtual Herbarium 2024), Flora of Pakistan (Eflora of Pakistan 2024), and several other prominent online herbaria (B, BM, BR, E, FI, FOB, G-DC, K, L, LINN, NYBG, P, TUB) were also analysed.

**Figure 1.**
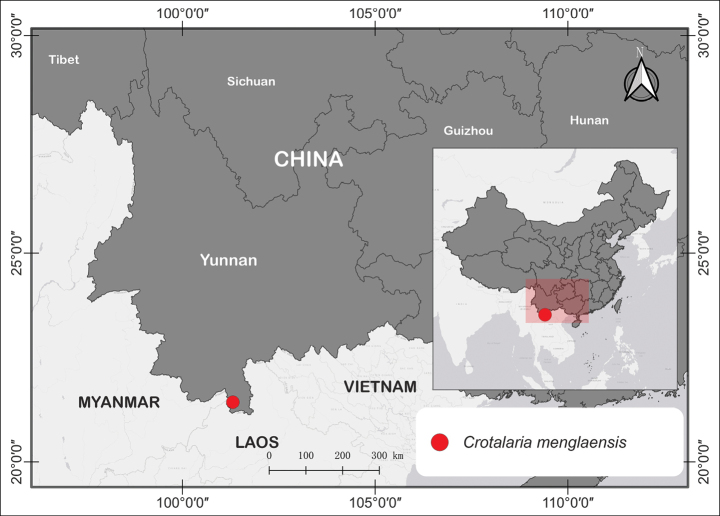
Map visualizing the only known occurrences of *Crotalariamenglaensis* S.A.Rather in Mengpeng village of Xishuangbanna Dai Autonomous Prefecture, Yunnan, China (red dot).

A distribution map was constructed to visualize the geographical distribution of the newly identified species. This map was developed with a foundational base map constructed from Natural Earth (www.naturalearthdata.com) and generated using QGIS version 3.28.2 (QGIS 2021) (Fig. 1).

### ﻿Taxa sampling for molecular study

Fresh and disease-free leaves were collected from specimens in the field and promptly dried using silica gel to facilitate subsequent DNA extraction. The voucher specimens were preserved at the Herbarium of Xishuangbanna Tropical Botanical Garden, Chinese Academy of Sciences (HITBC), and detailed information about each sample is provided in Suppl. material 1. The analysis included a total of 81 accessions, covering both the ITS region and the plastid marker *mat*K. Additionally, this dataset included two outgroup sequences from *Bolusiaamboensis* and *Euchlorahirsuta*, as well as nine publicly available sequences of African *Crotalaria* sourced from the NCBI databases (https://www.ncbi.nlm.nih.gov). Overall, our dataset comprised 81 individuals, representing 56 distinct *Crotalaria* species (Suppl. material 1).

### ﻿DNA Extraction, PCR Amplification and Sequencing

Genomic DNA was isolated using a DNeasy Plant Mini Kit (Qiagen, Amsterdam, The Netherlands) following the manufacturer’s protocol. The DNA quantity was confirmed via 0.8% agarose gel electrophoresis, and its concentration was determined using a SmartSpec^TM^ Plus Spectrophotometer (Bio-Rad, Hercules, CA, United States). Before amplification, the DNA samples were stored at -20 °C. Polymerase chain reactions (PCR) were performed in a 25 µL reaction volume comprising 2.5 µL of 10× buffer with 2 mM MgCl2, 1 U of Taq DNA polymerase, 1 µL of dNTPs (0.125 mM), 1 µL of each primer (5 pM), and 30–50 ng of total DNA. Nuclease-free water was added to reach the final volume. The optimal PCR conditions and detailed primer information are listed in Suppl. material 2. PCR products were visualized by electrophoresis on 0.8% agarose gels, followed by purification using BioMed multifunctional DNA fragment purification recovery kits (Beijing, China). The purified products were sequenced using the same primers used for PCR amplification. Sequencing was conducted on an ABI 3730 automated sequencer at Sangon Biotech, Shanghai, China.

### ﻿Sequence alignment and data analysis

To ensure the accuracy and authenticity of the sequences, the original trace files were subjected to rigorous validation through web-based BLASTn searches on the NCBI platform. We conducted sequence alignment in Geneious version 8.1.7, which included trimming, visual inspection, and manual adjustments (Kearse et al. 2012). The trimming parameters were set to an error probability of 0.1 per base and a quality threshold of 20, allowing the removal of any low-quality base calls at the 5’ and 3’ ends of the sequenced PCR products. Each gene was aligned separately using MUSCLE (Edgar 2004) within Geneious. To improve alignment quality and accuracy, ambiguous regions were trimmed using Gblocks v0.91b (Castresana 2000). Individual alignments were then concatenated to create a two-gene alignment for all 81 samples. Microsatellite repeats were excluded, and gaps were considered as missing data. Phylogenetic analyses were performed using the standard-maximum-likelihood (ML) method with IQ-TREE (Nguyen et al. 2015), Bayesian analysis with MrBayes (Ronquist et al. 2012), and the optimal nucleotide substitution model was determined with ModelFinder (Kalyaanamoorthy et al. 2017). Branch support in the ML tree was assessed through 10,000 ultrafast bootstrap replicates (Minh et al. 2013). All procedures were executed using PhyloSuite v1.2.2 (Zhang et al. 2020). The nrITS region of the newly discovered species yielded a 715 bp sequence, while the *mat*K region produced a 775 bp sequence. Concatenating these sequences generated a 1490 bp sequence-aligned matrix, with a total of 1690 characters across 81 accessions. The new species, *C.menglaensis*, differed from *C.incana* and *C.bracteata* by 12 nucleotide substitutions and one inversion at site 601 in both the ITS and *mat*K regions. Additionally, it showed three insertions, with lengths of 3 and 12 at sites 420–422 and 795–806, respectively, compared to its closest relatives. The multiple sequence alignment was submitted to TreeBASE with ID 31180.

### ﻿Morphometric analyses

To assess potential differences between the new species and their closest relatives and to determine which traits were most relevant for their identification, we conducted a principal component analysis (PCA) using the “factoextra” package in R version 4.3.0 (Kassambara and Mundt 2020; R Core Team 2023) with a significance level set at 5%. We examined three to five specimens of *C.menglaensis* S.A.Rather, *C.incana* L., and *C.bracteata* Roxb. ex DC. The length and width of leaflets, flowers, standards, wings, keels, seeds and pods were measured (Suppl. material 3). Correlation analysis was performed to eliminate highly correlated traits (r > 0.71) using the “corrplot” package in R version 4.3.0 (Wei and Simko 2021; R Core Team 2023). In total, four traits were retained for the PCA biplot analysis: keel length (KL), standard width (SW), seed width (SEW), and seed length (SEL) (Suppl. material 3).

## ﻿Results and discussion

The proposed new species, *Crotalariamenglaensis* S.A.Rather, resembles *C.incana* L. and *C.bracteata* Roxb. Ex DC. However, it differs from the former in several aspects. It has an ovate to oblanceolate leaflet shape with a pubescent leaf surface, an obovate-orbicular standard shape, a straight keel beak, and an elliptical to oblong pod shape. It differs from the latter in having a stem surface covered with white hairs, a pilose bract surface, a notched standard apex, planar callosity, an angled keel shape, and a tomentose pod indumentum. A comprehensive morphological comparison is presented in Table 1 to elucidate the distinctions between the new taxon and its closest relatives.

**Figure 2.**
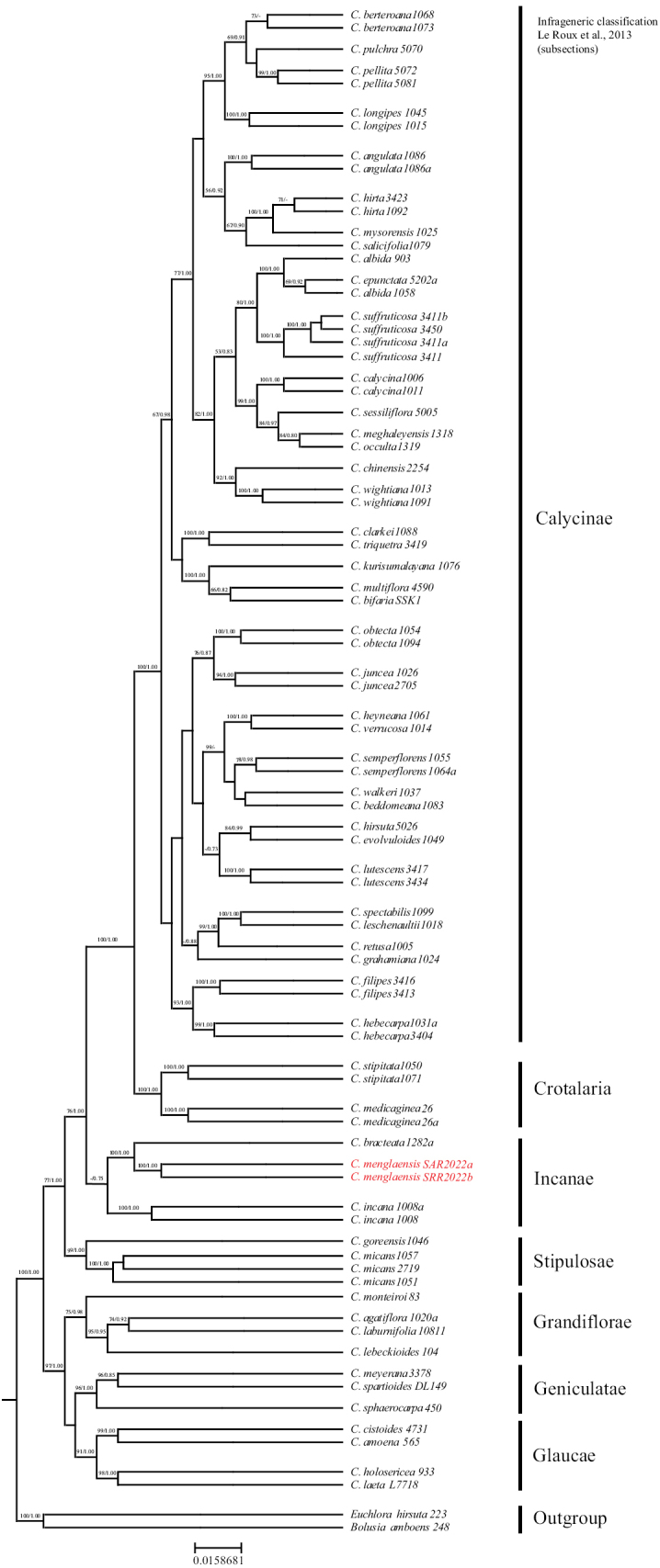
Phylogenetic hypothesis of the genus *Crotalaria* based on the concatenated matrix including *mat*K and nrITS sequences constructed via maximum likelihood as implemented in IQ Tree. Bootstrap values are printed above the branches. Since Bayesian analyses resulted in almost the same topology, only the ML tree made is presented here. The new species *Crotalariamenglaensis* S.A.Rather was marked in red. The names on the right side of the phylogeny correspond to the infrageneric classification of the genus *Crotalaria* by Le Roux et al. (2013).

The maximum likelihood (ML) and Bayesian tree phylogenies showed congruent topologies (Fig. 2). The phylogenetic tree identified seven major clades, corresponding to seven of the 11 sections proposed by Le Roux et al. (2013). These seven major clades (i.e., Calycinae, Crotalaria, Incanae, Stipulosae, Grandiflorae, Geniculatae and Glaucae) had bootstrap values greater than 80%. These clades are consistent with previous phylogenetic analyses (Subramaniam et al. 2013; Rather et al. 2018). Furthermore, phylogenetic analysis strongly supported the monophyletic status of the genus (100% BS). The phylogeny places the newly discovered species *C.menglaensis* S.A.Rather within a separate clade, supporting its distinction from other allied species included (Fig. 2). *C.menglaensis* S.A.Rather forms a distinct clade with *C.bracteata* Roxb. Ex DC. And *C.incana* L. (100% BS). Additionally, *C.menglaensis* S.A.Rather and *C.bracteata* Roxb. ex DC. form a sister clade with strong support (100% BS), and in turn, they are sisters of *C.incana* L. (100% BS) (Fig. 2).

**Figure 3.**
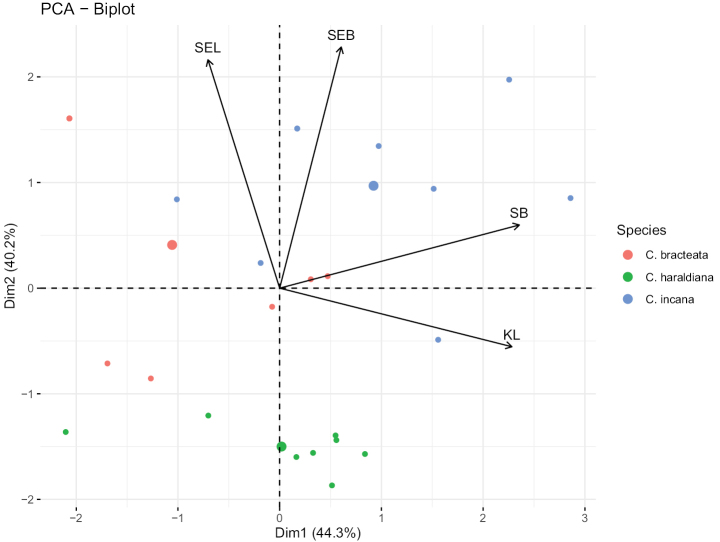
Scatter plot visualizing Dim1 and Dim2 from the principal component analyses based on the assembled morphological trait variables and accessions of the three species nested in the Incanae clade (see Fig. 2), namely, *C.incana* L., *C.bracteata* Roxb. ex DC. and the new species *Crotalariamenglaensis* S.A.Rather. Dim1 explained 44.3% of the variation, whereas DIM2 explained 40.2%. The vectors corresponded to KL = keel length, SW - standard width, SEW - seed width, and SEL - seed length.

Morphometric analyses based on principal component analysis using Pearson’s coefficient were employed to identify significant morphological characteristics that facilitated differentiation between the new species and its closest relatives based on gross morphology (Fig. 3, Suppl. material 4, Table 2). These morphometric analyses have proven highly valuable for elucidating correlations among variables or distances among groups and assessing the significance of each character. Fig. 3 illustrates the significant characteristic ratios that contribute to the uniqueness of the new species. Following Pearson’s correlation analysis, highly correlated traits were excluded, and four traits were used for statistical analysis (Fig. 3, Suppl. material 4, Table 2). The results showed significant differences in morphological characters, including keel length (KL), standard width (SW), seed width (SEW), and seed length (SEL), compared to those of allied species (Fig. 3). Dimension 1 of the PCA explained the greatest variance, followed by dimensions 2, 3, and 4, which collectively accounted for 84.48% of the variation, a substantial proportion (Fig. 3, Suppl. material 4, Table 2).

**Table 2.** Variance in the contributions of morphological trait variables as determined by principal component analysis.

Dimensions	Eigenvalue	Variance	Cumulative Variance Percent (%)
1	1.77	44.28	44.28
2	1.61	40.20	84.48
3	0.43	10.66	95.14
4	0.19	4.86	100.00

### ﻿Taxonomic treatment

#### ﻿ Crotalaria menglaensis

Taxon classificationPlantaeFabalesFabaceae

S.A. Rather sp. nov.

9C06759F-2FE8-581B-9FBA-ADED8DAAA956

urn:lsid:ipni.org:names:77343398-1

Fig. 4

##### Type.

China. Yunnan: Xishuangbanna Dai Autonomous Prefecture, Mengla County, Mengpengzhen., 21°26'57.42"N, 101°18' 31.49"E, alt. 577 m, 23 November 2022, SAR 202305 (holotype HITBC! isotypes KIB! PE! DUH! CAL!).

##### Diagnosis.

The new species is similar to two sympatrically occurring species, *C.incana* L. and *C.bracteata* Roxb. ex DC. However, *C.menglaensis* S.A.Rather differs from the former and latter in its height, 0.5 m (*vs* 1 *vs* 60–1.20); stem surface, pubescent with white hairs (*vs* pubescent brownish *vs* densely brownish yellow); bract surface, pilose (*vs* glabrous *vs* glabrous); leaflet shape, ovate to oblanceolate (*vs* elliptic obovate, or suborbicular *vs* narrowly elliptic) ; leaflet surface, pubescent (*vs* glabrous *vs* sparsely pilose); standard shape, obovate-orbicular (*vs* elliptic *vs* oblong); planar callosities (*vs* ridge *vs* ridge); keel shape, angled (*vs* subangled *vs* subangled); keel beak, straight (*vs* spirally twisted up to 90° *vs* slightly incurved); pod shape, elliptic to oblong (*vs* fusiform *vs* ellipsoid-fusiform); and pod indumentum tomentose (*vs* rusty pilose *vs* densely rusty pubescent).

**Figure 4.**
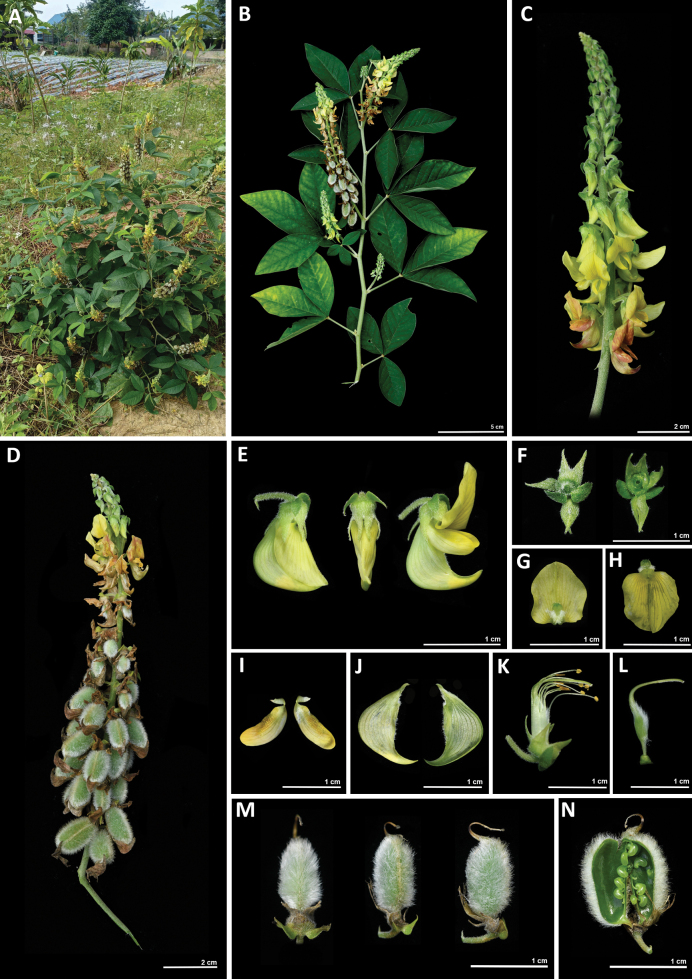
*Crotalariamenglaensis* S.A.Rather **A** habit **B** plant twigs with leaves and flowers **C** inflorescence with flowers **D** inflorescences with flowers and fruits **E** flower in dorsal, lateral, and ventral views **F** calyx showing the dorsal and ventral surfaces **G** standard adaxial surface **H** standard abaxial surface with paired planar callosity pairs at the base with white silky pubescence **I** wing petals with prominent cavae and a distinct claw **J** adaxial and abaxial surfaces of keel petals, beak not twisted, pubescence along the margins from the middle to the base of the keel petal **K** anthers monodelphous, 10 dimorphic anthers (common to all the species within the genus) **L** gynoecium showing the ovary, style, and stigma **M** pod in ventral, dorsal, and lateral views **N** pod splitted longitudinally with young seeds.

##### Description.

Stiff and erect herbs, ca. 0.5 m tall. Stems terete and densely pubescent. Stipules acicular. Leaves trifoliolate, alternate, petiole up to 30 mm long, lamina ovate to oblanceolate, 30–80 × 21–31 mm, terminal leaflet larger than the lateral ones, attenuated at the base, acute at apex, margin entire with puberulent indumentum, adaxial surface glabrescent, abaxial surface pubescent. Inflorescence a terminal or axillary raceme, a terminal raceme 80–120 mm bearing up to 12 flowers, and an axillary raceme 110–170 mm bearing up to 47 florets. Flower 10–11.9 × 3.3–4 mm. Bract lanceolate, 1.2–2 × 0. 6–0.7 mm covered with white pilose hairs inserted at the base of a pedicel. Pedicel ca. 4.7 mm, pubescent, reflexed downwards; bracteole ovate to obovate with an asymmetric base, 2.7–3.1 × 1.6–1.8 mm, hirsute, margin entire. Calyx 5-lobed, calyx tube ca. 2.4 × 2.9 mm, oblong-lanceolate, 2.2–2.9 × 0.4–0.71 mm, apex attenuate, densely ciliate along margins. Corolla primrose or strongly pale yellow, exserted beyond calyx, obovate-orbicular, ca. 8.8 × 7.4 mm, claw ca. 1.4 mm, with paired planar callosites at the base, ca. 0.6–0. 7 × 0. 7–0.8 mm; wing petals 7.1–7.3 × 2.3–2.9 mm, claw 1.52–1.84 × 6.3–0.77 mm, cavae 4.2–4.4 mm; keel angled, curvature below the middle, claw 3.4–3.6 × 1.2–1.4 mm, glabrous, beak straight. Staminal sheath 7.8 mm; filaments free, glabrous, shorter filament 3.7–6.7 mm, longer filament 7.7–8.0 mm; anthers dimorphic, basifixed ones longer, ensiform, ca. 1.2–1.5 mm, dorsifixed ones shorter, orbicular ca. 0.5–0.6 mm. Ovary sessile, linear, ca. 3.3 × 1.5 mm, inflated, style 8.2 mm long, geniculate, trichomes in a single row; stigma brush-like and contracted, ca. 0. 21 mm long, hairy. Pods elliptic to oblong, 14.2–15 × 6–7.7 mm, tomentose, with persistent style. Seeds 2.2–2.5 × 0.9–1.2 mm, bright citrine, smooth and glossy.

##### Phenology.

The plants were observed to bear flowers and fruits from October to January.

##### Etymology.

The specific epithet of the new species “*menglaensis*” is derived from the type locality of this species.

##### Distribution and habitat.

*Crotalariamenglaensis* S.A. Rather is found in grasslands and exposed areas of Mengpeng, Mengla County, within the Xishuangbanna Dai Autonomous Prefecture, Yunnan, China.

##### Uses.

Locals use the pods of this species as a food source. Additionally, its roots and seeds are utilized in traditional medicine to treat various digestive disorders.

##### IUCN Red List Category.

This species is exclusively documented in a single location where clustered populations of fewer than 100 mature individuals have been observed. Its habitat is adjacent to roads and agricultural land and is consistently affected by anthropogenic activities such as grazing, deforestation, cultivation, and landscape management. The potential degradation of its natural habitat and restricted geographical range significantly threatens its survival. Therefore, according to the IUCN Standards and Petitions Committee (2019), this species should be considered critically endangered under criteria A4, B2a, C2a, and D1. These criteria denote species facing a very high risk of extinction in the wild.

##### Additional specimens examined

**(paratypes). China, Yunnan**. Mengla, in forest, alt. 1600 m, 12 June 2012, *Y.M. Shi & W.S. Chen 254655* (KUN). Xishuangbanna, Mengla, in the forest, 1650 m, 16 July 2014, *Y.M. Shui & W.S. Chen 245266* (KUN). Xishuangbanna, Mengla, in the forest, 1450 m, 14 August 2016, *Z.Y Wen & Z.A Wang 524694* (KUN). Hekou, on forest edges, 1459 m, 25 November 2005, *Z.Y. Chang et al. 162458* (KUN). Xishuangbanna, Mengla, in grasslands, 1200 m, 3 August 2007, *Z. Y. Chang 445123* (KUN). Xishuangbanna, Mengla, 1180 m, 25 August 2010, *Z.Y. Chiang 2005387* (HITBC).

## Supplementary Material

XML Treatment for Crotalaria menglaensis

## ﻿Additional information

### Author ORCIDs

Shabir A. Rather https://orcid.org/0000-0002-0356-275X

Sirilak Radbouchoom https://orcid.org/0000-0002-6027-7832

Kaikai Wang https://orcid.org/0000-0002-0035-2466

Yunxue Xiao https://orcid.org/0000-0003-2997-651X

Hongmei Liu https://orcid.org/0000-0002-0780-308X

Harald Schneider https://orcid.org/0000-0002-4548-7268

### Data availability

All of the data that support the findings of this study are available in the main text or Supplementary Information.

## Supplementary materials

Supplementary material 1 Plant accessions used for the molecular analysis of *Crotalaria* along with their GenBank accession numbers Data type doc https://binary.pensoft.net/file/1066593This dataset is made available under the Open Database License (http://opendatacommons.org/licenses/odbl/1.0/). The Open Database License (ODbL) is a license agreement intended to allow users to freely share, modify, and use this Dataset while maintaining this same freedom for others, provided that the original source and author(s) are credited.Shabir A. Rather, Sirilak Radbouchoom, Kaikai Wang, Yunxue Xiao, Hongmei Liu, Harald Schneider

Supplementary material 2 Details of primers used for ampliﬁcation and subsequent sequencing in the present study Data type doc https://binary.pensoft.net/file/1066594This dataset is made available under the Open Database License (http://opendatacommons.org/licenses/odbl/1.0/). The Open Database License (ODbL) is a license agreement intended to allow users to freely share, modify, and use this Dataset while maintaining this same freedom for others, provided that the original source and author(s) are credited.Shabir A. Rather, Sirilak Radbouchoom, Kaikai Wang, Yunxue Xiao, Hongmei Liu, Harald Schneider

Supplementary material 3 The morphological traits of *Crotalariamenglaensis* S.A.Rather. and its close relatives *C.bracteata* Roxb. ex DC. and *C.incana* L. Data type docx https://binary.pensoft.net/file/1066595This dataset is made available under the Open Database License (http://opendatacommons.org/licenses/odbl/1.0/). The Open Database License (ODbL) is a license agreement intended to allow users to freely share, modify, and use this Dataset while maintaining this same freedom for others, provided that the original source and author(s) are credited.Shabir A. Rather, Sirilak Radbouchoom, Kaikai Wang, Yunxue Xiao, Hongmei Liu, Harald Schneider

Supplementary material 4 Pearson correlation analysis of 14 morphological traits of *Crotalariamenglaensis* S.A.Rather. Data type tif https://binary.pensoft.net/file/1066596This dataset is made available under the Open Database License (http://opendatacommons.org/licenses/odbl/1.0/). The Open Database License (ODbL) is a license agreement intended to allow users to freely share, modify, and use this Dataset while maintaining this same freedom for others, provided that the original source and author(s) are credited.Shabir A. Rather, Sirilak Radbouchoom, Kaikai Wang, Yunxue Xiao, Hongmei Liu, Harald Schneider
