# Supplementary material for: ﻿Molecular, morphological, and morphometric evidence reveal a new, critically endangered rattlepod (Crotalaria, Fabaceae/Leguminosae, Papilionoideae) from tropical China
Source: PhytoKeys. 2024 Jun 11;242:333–48. doi: 10.3897/phytokeys.242.122407 (PMC11188087; doi:10.3897/phytokeys.242.122407)
Supplement: Supplementary material 1 — Plant accessions used for the molecular analysis of Crotalaria along with their GenBank accession numbers [file phytokeys-242-333_article-122407__-s001.doc]

| **Taxon** | **Voucher (Herbarium)** | **Locality** | **ITS** | ***mat*K** |
| --- | --- | --- | --- | --- |
| *Crotalaria agatiflora* Schweinf. | Subramaniam 1020a | India, Tamil Nadu, Kodaikanal | JN99021 | Submitted |
| *C. albida* Heyne ex Roth | Subramaniam 903 | India, Uttarakhand, Dehradun | JN990126 | Submitted |
| *C. albida* Heyne ex Roth | Subramaniam 1058 | India, Tamil Nadu, Coonoor | JN990120 | Submitted |
| *C. angulata* Mill. | Subramaniam 1086 | India, Tamil Nadu, Coimbatore | JQ945951 | Submitted |
| *C. angulata* Mill. | Subramaniam 1086a | India, Tamil Nadu, Coimbatore | KP698615 | Submitted |
| *C. beddomeana* DC. | Subramaniam 1083 | India, Kerala, Munnar | JQ945935 | Submitted |
| *C. bifaria* Benth. | Sharad Kamble SSK-1 | India, Karnataka, Badami | JQ945936 | Submitted |
| *C. berteroana* DC. | Subramaniam 1073 | India, Kerala, Muthanga | KR673341 | Submitted |
| *C. berteroana* DC. | Subramaniam 1068 | India, Kerala, Muthanga | Submitted | Submitted |
| *C. bracteata* DC | SAR 1282 | China, Yunnan, Xishuangbanna, Mengyan | KX390776 | Submitted |
| *C. bracteata* DC | SAR 1282 | China, Yunnan, Xishuangbanna, Mengyan | KX371676 | Submitted |
| *C. calycina* Schrank | Subramaniam 1006 | India, Tamil Nadu, Palani Hills | KP698617 | Submitted |
| *C. calycina* Schrank | Subramaniam 1011 | India, Tamil Nadu, Palani Hills | JN990122 | Submitted |
| *C. clarkei* Gamble | Subramaniam 1088 | India, Kerala, Wagamon | JQ945932 | Submitted |
| *C. evolvuloides* Benth. | Subramaniam 1049 | India, Tamil Nadu, Palani Hills | KP698639 | Submitted |
| *C. epunctata* Dalz. | Manudev KM 5202a | India, Odisha, Khandamal district | JQ945952 | Submitted |
| *C. filipes* Benth. var. *trichophora* (Benth. ex Baker) Cook. | Subramaniam and Pandey 3413 | India, Maharashtra, Kolhapur | JQ945925 | Submitted |
| *C. filipes* var. *filipes* Benth. | Subramaniam and Pandey 3416 | India, Maharashtra, Kolhapur | JQ945926 | Submitted |
| *C. goreensis* Guill. & Perr. | Subramaniam 1046 | India, Karnataka, Bengaluru, Yelahanka | JN990128 | Submitted |
| *C. grahamiana* Wight & Arn. | Subramaniam 1024 | India, Tamil Nadu, Pazhani hills | JN990129 | Submitted |
| *C. hirta* Willd. | Subramaniam 1092 | India, Tamil Nadu, Coimbatore | JQ945928 | Submitted |
| *C. hirta* Rottl. ex Willd. | Subramaniam Pandey 3423 | India, Maharashtra, Kohlapur | JQ945929 | Submitted |
| *C. hirsuta* Willd. | Subramaniam and Pandey 5026 | India, Himachal Pradesh | KP698652 | Submitted |
| *C. hebecarpa* (DC.) Rudd | Subramaniam 1031a | India, Tamil Nadu, Theni | JN990130 | Submitted |
| *C. hebecarpa* (DC.) Rudd | Subramaniam 3404 | India, Maharashtra, Kolhapur | KP698610 | Submitted |
| *C. heyneana* Wight & Arn. | Subramaniam 1061 | India, Kerala, Thamarassery pass | JQ955942 | Submitted |
| *C. incana* L. | SAR 1008 | China, Yunnan, Xishuangbanna, Mengpengzhen | JN990131 | Submitted |
| *C. incana* L. | SAR 1008a | China, Yunnan, Xishuangbanna, Mengpengzhen | JN990133 | Submitted |
| *C. juncea* L. | Subramaniam 1026 | India, Tamil Nadu, Kodaikanal | JN990138 | Submitted |
| *C. juncea* L. | Rather Pandey 2705 | India, Meghalaya, Nehu Campus | Submitted | Submitted |
| *C. kurisumalayana* Sibichen & Nampy | Subramaniam 1076 | India, Kerala, Kurisumalay | KP698650 | Submitted |
| *C. Leschenaulti* DC. | Subramaniam 1018 | India, Tamil Nadu, Palani Hills | JN990118 | Submitted |
| *C. lutescens* Dalz. | Subramaniam and Pandey 3434 | India, Maharashtra, Kolhapur | JQ945934 | Submitted |
| *C. lutescens* Dalz. | Subramaniam and Pandey 3417 | India, Maharashtra, Kohlapur | JQ945933 | Submitted |
| *C. longipes* Wight & Arn. | Subramaniam 1015 | India, Tamil Nadu, Palani Hills, Perumaalmalai | KP698626 | Submitted |
| *Crotalaria longipes* Wight & Arn. | Subramaniam and Pandey 1045 | India, Tamil Nadu, Shevaroy Hills | JN990113 | Submitted |
| *C. meghalayensis* Danda and Pandey | Danda and Pandey 1318 | India, Meghalaya, Jowai | KR059588 | Submitted |
| *C. medicaginea* var. *luxurians* (Benth.) Baker | Subramaniam 26 | India, Uttar Pradesh, Ghaziabad | JN990115 | Submitted |
| *C. medicaginea* var. *luxurians* (Benth.) Baker | Subramaniam 26a | India, Uttar Pradesh, Ghaziabad | KP698668 | Submitted |
| *C. micans* Link | Subramaniam 1051 | India, Tamil Nadu, Shevaroy Hills | KP698660 | Submitted |
| *C. micans* Link | Subramaniam 1057 | India, Tamil Nadu, Shevaroy Hills | KP698659 | Submitted |
| *C. micans* Link | Rather and Pandey 2719 | India, Arunachal Pradesh, Doimukh | Submitted | Submitted |
| *C. mysorensis* Roth | Subramaniam 1025 | India, Tamil Nadu, Kodaikanal | JN990114 | Submitted |
| *C. obtecta* Wight & Arn. var. *obtecta* | Subramaniam 1054 | India, Tamil Nadu, Coonoor | KP698612 | Submitted |
| *C. obtecta var. glabrescens* (Benth.) Baker | Subramaniam 1094 | India, Kerala, Munnar | JQ945950 | Submitted |
| *C. occulta* Grah. ex Benth. | Shagun and Pandey 1319 | India, Meghalaya, Nehu Campus | KR095590 | Submitted |
| *C. pellita* DC. | Subramaniam and Pandey 5072 | India, Andhra Pradesh, SK University campus | KP698632 | Submitted |
| *C. pellita* DC. | Subramaniam and Pandey 5081 | India, Tamil Nadu, Shevaroy Hills | KP698633 | Submitted |
| *C. pulchra* Andr. | Subramaniam and Pandey 5070 | India, Karnataka, Siddara Betta | KX371741 | Submitted |
| *C. retusa* L. | Subramaniam 1005 | India, Delhi, Delhi University Botanical Garden | KP698625 | Submitted |
| *C. semperflorens* Vent. | Subramaniam 1055 | India, Tamil Nadu, Theni | KP698674 | Submitted |
| *C. semperflorens* Vent. | Subramaniam 1064a | India, Tamil Nadu, Theni | JQ945940 | Submitted |
| *C.sessiliflora var sessiliflora* f. garhwalensis L. | Subramaniam 5005 | India, Himachal Pradesh, Naahan | KP698619 | Submitted |
| *C. spectablis* Roth | Subramaniam and Pandey 1099 | India, Delhi, Delhi University Botanical Garden | JN990112 | Submitted |
| *C. stipitata* Wight & Arn. | Subramaniam 1050 | India, Tamil Nadu, Ootacamund | JN990116 | Submitted |
| *C. stipitata* Wight & Arn. | Subramaniam 1071 | India, Tamil Nadu, Ootacamund | KP698669 | Submitted |
| *C. suffruticosa* Subramaniam & Pandey | Subramaniam and Pandey 3411 | India, Maharashtra, Kolhapur, | KY321453 | Submitted |
| *C. suffruticosa* Subramaniam & Pandey | Subramaniam and Pandey 3411a | India, Maharashtra, Kolhapur, | KY321454 | Submitted |
| *C. suffruticosa* Subramaniam & Pandey | Subramaniam and Pandey 3411b | India, Maharashtra, Kolhapur, | KY321455 | Submitted |
| *C. suffruticosa* Subramaniam & Pandey | Subramaniam and Pandey 3450 | India, Maharashtra, Kolhapur, | KY321456 | Submitted |
| *C. salicifolia* Wight & Arn. | Subramaniam 1079 | India, Kerala, Kurisumalay | JQ945948 | Submitted |
| *C. triquetra* Dalz. | Subramaniam Pandey 3419 | India, Maharashtra, Kohlapur | JQ945930 | Submitted |
| *C. verrucosa* L. | Subramaniam 1014 | India, Tamil Nadu, Palani Hills | KP698645 | Submitted |
| *C. walkeri* Arn. | Subramaniam 1037 | India, Tamil Nadu | JN990111 | Submitted |
| *C. wightiana* Wight & Arn. | Subramanim 1013 | India, Tamil Nadu, Pazhani hills | JQ945947 | Submitted |
| *C. menglaensis* S.A.Rather | SAR2022a | Yunnan, China, Xishuangbanna, Mengpengzhen | Submitted | Submitted |
| *C. menglaensis* S.A.Rather | SAR2022b | Yunnan, China, Xishuangbanna, Mengpengzhen | Submitted | Submitted |
| *C. wightiana* Wight & Arn. | Subramaniam 1091 | India, Kerala, Munnar | JQ945954 | Submitted |
| *C. sphaerocarpa* DC. | Schutte 450 | South Africa, Limpopo | JQ067139 | JQ041106 |
| *C. multiflora* Arn. | Hepper 4590 | Sri Lanka, Uva | JQ067336 | - |
| *C. chinensis* L. | Sorensen et al 2254 | Thailand, Loei | JQ067335 | - |
| *C. laburnifolia subsp petiolaris* (Franch.) Polhill | Thulin 10811 | Somalia, Sanaag | JX120578 | JX120589 |
| *C. lebeckioides* Bond | Le Roux et al 104 | South Africa, Western Cape | JQ067321 | JQ067568 |
| *C. cistoides* DC. | Bidgood et al 4731 | Thailand, Kanchanaburi | JQ067188 | JQ041009 |
| *C. meyerana* Steud. | Williamson 3378 | Namibia, Karas | JQ067136 | JQ041078 |
| *C. spartioides* DC. | Lutombi DL149 | Namibia, Hardap | JQ067149 | JQ041105 |
| *C. laeta* Mart. ex Benth. | Coradiv L7718 | Brazil, Ceara | JQ067325 | JQ067571 |
| *C. holosericea* Nees & Mart. | Lewis et al 933 | Brazil, Bahia | JQ067343 | JQ067572 |
| *C. monteiroi* Baker f. | Schutte 83 | South Africa, KwaZulu Natal | JQ067129 | JQ041083 |
| *Euchlora hirsuta* (Thunb.) Druce | Boatwright 223 | South Africa, Northern Cape | EU347879 | JQ041113 |
| *Bolusia amboensis* (Schinz) Harms | Boatwright 248 | South Africa, Northern Cape | EU347881 | JQ040984 |
