# Supplementary material for: ﻿Molecular, morphological, and morphometric evidence reveal a new, critically endangered rattlepod (Crotalaria, Fabaceae/Leguminosae, Papilionoideae) from tropical China
Source: PhytoKeys. 2024 Jun 11;242:333–48. doi: 10.3897/phytokeys.242.122407 (PMC11188087; doi:10.3897/phytokeys.242.122407)
Supplement: Supplementary material 2 — Details of primers used for ampliﬁcation and subsequent sequencing in the present study [file phytokeys-242-333_article-122407__-s002.doc]

| **Region** | **Primers** | **Sequence** | **PCR conditions** | **Reference** |
| --- | --- | --- | --- | --- |
| ITS | ITS F | ATGCGATACTTGGTGTGAAT |  | Sun et al., 1994 |
|  | ITS R | GACGCTTCTCCAGACTACAAT | 94°C-4 min, (94°C-30 s, 53°C-40 s, 72°C-40 s) 35 cycles and final extension with 72°C-7 min |  |
| *mat*K | 3F_KIM f | CGT ACA GTA CTT TTG TGT TTA CGA G | 95°C-4 min, (95°C-30 s, 50°C-40 s, 72°C-50 s) 35 cycles and final extension with 72°C-2 min | Ki-Joong Kim 2008 |
|  | 1R_KIM r | ACC CAG TCC ATC TGG AAA TCT TGG TTC |  |  |
