# Supplementary material for: ﻿Molecular, morphological, and morphometric evidence reveal a new, critically endangered rattlepod (Crotalaria, Fabaceae/Leguminosae, Papilionoideae) from tropical China
Source: PhytoKeys. 2024 Jun 11;242:333–48. doi: 10.3897/phytokeys.242.122407 (PMC11188087; doi:10.3897/phytokeys.242.122407)
Supplement: Supplementary material 3 — The morphological traits of Crotalariamenglaensis S.A.Rather. and its close relatives C.bracteata Roxb. ex DC. and C.incana L. [file phytokeys-242-333_article-122407__-s003.docx]

| **Species** | **FL** | **FW** | **SL** | **SW** | **WL** | **WW** | **KL** | **KW** | **SEL** | **SEW** | **LL** | **LW** | **PL** | **PW** |
| --- | --- | --- | --- | --- | --- | --- | --- | --- | --- | --- | --- | --- | --- | --- |
| *C. menglaensis* | 1 | 0.3 | 0.9 | 0.7 | 0.7 | 0.2 | 0.3 | 0.1 | 0.22 | 0.09 | 3 | 2.1 | 1.42 | 0.6 |
| *C. menglaensis* | 1.2 | 0.4 | 0 | 0 | 0.7 | 0.3 | 0.4 | 0.1 | 0.25 | 0.12 | 8 | 3.1 | 1.5 | 0.8 |
| *C. menglaensis* | 1.2 | 1.4 | 1 | 0.6 | 0.7 | 0.2 | 1.1 | 0.4 | 0.2 | 0.1 | 5 | 2 | 0.6 | 0.4 |
| *C. menglaensis* | 1.5 | 1.5 | 0.9 | 0.6 | 0.9 | 0.2 | 1.2 | 0.4 | 0.2 | 0.12 | 9 | 4.5 | 1.2 | 0.8 |
| *C. menglaensis* | 1 | 1.2 | 1 | 0.6 | 0.8 | 0.3 | 1.3 | 0.5 | 0.15 | 0.1 | 8 | 4 | 1.2 | 0.7 |
| *C. menglaensis* | 1.2 | 1.5 | 1.2 | 0.8 | 0.8 | 0.3 | 1.3 | 0.5 | 0.2 | 0.1 | 8.5 | 3.2 | 1.3 | 0.8 |
| *C. menglaensis* | 1.2 | 1 | 1.2 | 0.7 | 0.8 | 0.3 | 1.2 | 0.5 | 0.2 | 0.15 | 8 | 3.5 | 1.2 | 0.7 |
| *C. menglaensis* | 1.3 | 1.2 | 1.3 | 0.6 | 0.8 | 0.3 | 1.4 | 0.5 | 0.25 | 0.12 | 7.3 | 3.6 | 1.2 | 0.8 |
| *C. incana* | 1 | 0.9 | 1 | 1.3 | 1.2 | 0.8 | 1.2 | 0.8 | 0.3 | 0.2 | 2.5 | 2.5 | 1.26 | 0.4 |
| *C. incana* | 1.5 | 1.2 | 1.2 | 1.1 | 1.2 | 0.9 | 1.1 | 0.6 | 0.6 | 0.4 | 3.6 | 3 | 1.69 | 0.6 |
| *C. incana* | 1.4 | 1 | 1.5 | 1.2 | 1.5 | 0.8 | 1.6 | 0.9 | 0.5 | 0.7 | 3 | 2.3 | 1.97 | 0.7 |
| *C. incana* | 1.4 | 0.8 | 1.7 | 1.4 | 1.4 | 0.6 | 1.8 | 0.5 | 0.4 | 0.5 | 4 | 3.1 | 1.86 | 0.7 |
| *C. incana* | 1.9 | 0.5 | 1.1 | 1.1 | 1.3 | 0.7 | 1.1 | 0.3 | 0.3 | 0.6 | 1.4 | 1.02 | 2.03 | 0.6 |
| *C. incana* | 1.1 | 0.4 | 0.8 | 0.8 | 1.1 | 0.4 | 0.6 | 0.5 | 0.4 | 0.3 | 0.3 | 1.45 | 1.38 | 0.3 |
| *C. incana* | 1 | 0.2 | 0.6 | 0.5 | 0.8 | 0.4 | 0.5 | 0.3 | 0.5 | 0.4 | 1.1 | 0.36 | 1.27 | 0.4 |
| *C. incana* | 0.6 | 0.9 | 1.1 | 1 | 0.6 | 0.3 | 0.5 | 0.6 | 0.4 | 0.6 | 2.3 | 0.71 | 1.71 | 0.6 |
| *C. incana* | 1.1 | 0.9 | 1.1 | 1 | 0.6 | 0.3 | 0.5 | 0.6 | 0.4 | 0.6 | 2.3 | 0.71 | 1.71 | 0.6 |
| *C. bracteata* | 1.2 | 0.5 | 0.8 | 0.8 | 0.8 | 0.3 | 1 | 0.5 | 0.35 | 0.36 | 2.1 | 0.95 | 0.55 | 0.2 |
| *C. bracteata* | 0.7 | 0.3 | 0.9 | 0.61 | 0.6 | 0.2 | 0.9 | 0.3 | 0.34 | 0.31 | 3.1 | 1.35 | 0.51 | 0.2 |
| *C. bracteata* | 0.7 | 0.5 | 0.6 | 0.7 | 0.6 | 0.4 | 0.8 | 0.4 | 0.2 | 0.5 | 1.8 | 0.85 | 0.6 | 0.5 |
| *C. bracteata* | 0.3 | 0.3 | 0.2 | 0.3 | 0.2 | 0.2 | 0.3 | 0.2 | 0.13 | 0.33 | 2.3 | 1.23 | 0.54 | 0.2 |
| *C. bracteata* | 0.3 | 0.3 | 0.3 | 0.3 | 0.2 | 0.3 | 0.3 | 0.3 | 0.3 | 0.2 | 2 | 0.66 | 0.53 | 0.3 |
| *C. bracteata* | 0.4 | 0.2 | 0.2 | 0.2 | 0.2 | 0.2 | 0.3 | 0.2 | 0.7 | 0.4 | 1.5 | 0.7 | 0.45 | 0.2 |
| *C. bracteata* | 0.3 | 0.2 | 0.2 | 0.2 | 0.2 | 0.2 | 0.3 | 0.2 | 0.7 | 0.4 | 2.3 | 0.92 | 0.75 | 0.4 |
| *C. bracteata* | 0.3 | 0.1 | 0.2 | 0.2 | 0.2 | 0.2 | 0.3 | 0.2 | 0.7 | 0.4 | 3.2 | 1.5 | 0.82 | 0.3 |
