# Supplementary figures and images for: ﻿Molecular, morphological, and morphometric evidence reveal a new, critically endangered rattlepod (Crotalaria, Fabaceae/Leguminosae, Papilionoideae) from tropical China
Source: PhytoKeys. 2024 Jun 11;242:333–48. doi: 10.3897/phytokeys.242.122407 (PMC11188087; doi:10.3897/phytokeys.242.122407)

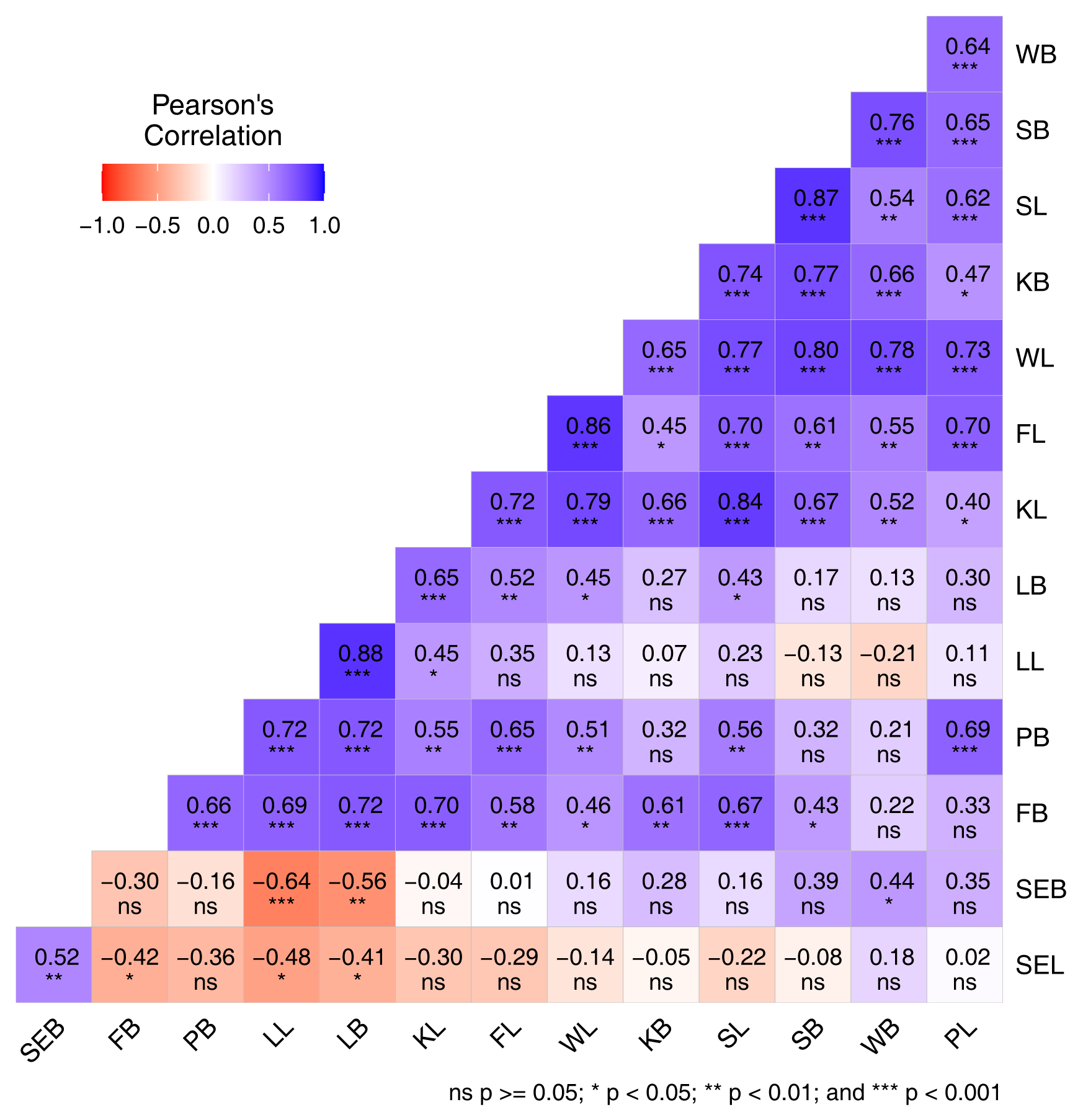

Supplement: Supplementary material 4 — Pearson correlation analysis of 14 morphological traits of Crotalariamenglaensis S.A.Rather. [file phytokeys-242-333_article-122407__-s004.tif]
